# The impact of inflammatory and metabolic markers on depression, anxiety, and cognition after COVID-19: a narrative review

**DOI:** 10.47626/2237-6089-2022-0599

**Published:** 2024-11-26

**Authors:** Elton Jorge Bessa Diniz, Fulvio Alexandre Scorza, Fabrício Maués Santos Rodrigues, Claudia Berlim de Mello, Tatiana Carvalho de Souza Bonetti, Karina Ramalho Bortoluci, Jair de Jesus Mari

**Affiliations:** 1 Universidade Federal de São Paulo Escola Paulista de Medicina Departamento de Psiquiatria e Psicologia Médica São Paulo SP Brazil Departamento de Psiquiatria e Psicologia Médica, Escola Paulista de Medicina (EPM), Universidade Federal de São Paulo (UNIFESP), São Paulo, SP, Brazil.; 2 UNIFESP EPM Departamento de Neurologia/Neurocirurgia São Paulo SP Brazil Disciplina de Neurociências, Departamento de Neurologia/Neurocirurgia, EPM, UNIFESP, São Paulo, SP, Brazil.; 3 UNIFESP EPM Departamento de Psicobiologia São Paulo SP Brazil Departamento de Psicobiologia, EPM, UNIFESP, São Paulo, SP, Brazil.; 4 UNIFESP EPM Departamento de Ginecologia São Paulo SP Brazil Departamento de Ginecologia, EPM, UNIFESP, São Paulo, SP, Brazil.; 5 UNIFESP EPM Departamento de Farmacologia São Paulo SP Brazil Departamento de Farmacologia, EPM, UNIFESP, São Paulo, SP, Brazil.

**Keywords:** COVID-19, inflammation, metabolomics, depression, anxiety, cognitive impairment

## Abstract

**Objectives:**

There has been growing concern about the long-term effects of coronavirus disease 2019 (COVID-19) on mental health. The biological factors common to psychiatric conditions and COVID-19 are not yet fully understood.

**Methods:**

We narratively reviewed prospective longitudinal studies that measured metabolic or inflammatory markers and assessed psychiatric sequelae and cognitive impairment in individuals with COVID-19 at least 3 months after infection. A literature search identified three relevant cohort studies.

**Results:**

Overall, depressive symptomatology and cognitive deficits persisted for up to 1 year after COVID-19; depression and cognitive changes were predicted by acute inflammatory markers, and changes in these markers correlated with changes in depressive symptomatology; female sex, obesity, and the presence of inflammatory markers were associated with more severe clusters of physical and mental health status in patients’ self-perceived recovery; and plasma metabolic profiles of patients continued to differ from those of healthy controls 3 months after hospital discharge, which were associated with widespread alterations in neuroimaging, reflecting issues with white matter integrity.

**Conclusion:**

In individuals affected by COVID-19, prolonged exposure to stress and alterations in metabolic and inflammatory markers play a central role in psychiatric sequelae and cognitive deficits in the long term.

## Introduction

In the last few decades, the burden of mental disorders has surpassed those of cancer and cardiovascular diseases on a global scale.^1^ The coronavirus disease 2019 (COVID-19) caused by severe acute respiratory syndrome coronavirus 2 (SARS-CoV-2) is expected to increase the prevalence of psychiatric disorders,^2^ yet the interactions between the biological, psychological, and social determinants of this relationship are not yet understood. Public health interventions and vaccination strategies have been implemented to control the dissemination of COVID-19, but it remains a serious global threat. Although it is predominantly a respiratory pathogen, SARS-CoV-2 can cause significant damage to multiple organ systems, including the nervous system, and is associated with significant neuropsychiatric morbidities.^3-5^

COVID-19 can lead to disability and death, causing increased generalized anxiety. This is in addition to the social isolation and distress caused by associated lockdowns, financial problems, and harmful media coverage.^6,7^ Moreover, the acute phase of the infection can be associated with psychiatric conditions including depression, anxiety, post-traumatic stress disorder (PTSD), suicide, self-harm, and alcohol and substance misuse.^2^ A meta-analysis found a 45% (95%CI 37-54%, I² = 96%) prevalence of depression, and 47% (95%CI 37-57%, I² = 97%) prevalence of anxiety in COVID-19 patients.^8^

During the acute phase of COVID-19, sufferers may experience a variety of neuropsychiatric symptoms, which are not always related to the severity of the disease.^9^ These include delirium, depression, anxiety, and insomnia.^10-12^ The underlying mechanisms can be a consequence of direct infection of the central nervous system (CNS) or indirect immune dysfunction or a nonspecific autoimmune response.^13^ Metabolites, as the downstream products of genes and proteins, provide a functional readout of cellular processes and are often used as biomarkers. Metabolomic approaches can reveal the specific processes occurring in affected tissue^14^ and changes in serum metabolites are associated with COVID-19 infection and its consequences.

Psychological stress resulting from facing a potentially fatal illness, social isolation, concerns about infecting others, and social stigma may also contribute to psychiatric symptoms. Exposure to a life-threatening illness can lead to symptoms of PTSD.^15,16^ Studies suggest that certain genetic markers can increase vulnerability to trauma-induced dysregulation of specific neuronal networks, potentially enabling the identification of those at high risk of PTSD during COVID-19 infection.^15^

The World Health Organization (WHO) warned of possible consequences of COVID-19 infection on cognition from the earliest months.^17-19^ Potential neurocognitive sequelae have been attributed to CNS insults due to neuroinflammatory responses, infection of vascular endothelial cells, and microinvasion of the CNS through the cranial nerves.^20,21^ It is now well recognized that COVID-19 infection may impact cognition.^22^ Consequently, researchers and clinicians have endeavored to characterize cognitive impairments in recovered individuals and to determine associations with disease severity and bidirectional relations with mental health conditions. This review aims to understand the impact of COVID-19 on mental health and cognition, and their association with metabolic and inflammatory markers. First, we will present the primary findings of this review, followed by a discussion on the key pathophysiological changes associated with COVID-19, including inflammation, metabolomics, cognition, and stress.

## Methods

An electronic search of the literature was performed in PubMed on October 30, 2022, using the following keywords: ([COVID-19 or SARS-CoV-2] and [depress* or anxiety or "traumatic experiences" or "posttraumatic stress disorder" or trauma or inflammat* or neuroendocrine or metabolomic or "cognitive impairment" or cognit* or "mental health" or "psychiatric disorder" or psychiatr* or neuropsychiatr*]). We included studies that: 1) had a prospective longitudinal design with at least 3 months of follow-up (to capture the long-term effects of biological markers on mental health and cognition after COVID-19 infection); and 2) correlated depression/anxiety and cognitive changes with biological markers (e.g., laboratory parameters to measure inflammation or metabolic alterations; neuroimaging alterations). We excluded studies that assessed mental health outcomes solely, without any biological parameters or cognitive assessment. We extracted the following data from the included studies: a) study location and design; b) characteristics of the population (number of individuals and mean age); c) timing of assessments and assessment tools used for mental health and cognition; d) biological parameters used; and e) the main findings regarding mental health status, cognitive functions, and biological parameters.

## Results

Three cohort studies were found that fulfilled our selection criteria. The first of these was a prospective case-control study from China that sought to understand the associations between metabolic and structural changes in the brain 3 months after COVID-19 infection and symptoms of anxiety and depression.^23^ The second study, from Italy, evaluated the role of inflammation in depression, anxiety, and cognitive changes 3 months after COVID-19 infection.^11^ The third study, from the UK, aimed to understand the relationships between patient-perceived recovery, mental and physical health status, and inflammatory profile in individuals discharged from the hospital after 1 year of COVID-19 infection.^24^ A summary of the three studies is presented in Table 1.

**Table 1:** Summaries of the studies reviewed

Study	Location	Design	Population	Time of assessment	Assessment tools	Findings
Mazza et al.^11^	San Raffaele Hospital, Milan, Italy	Single-center prospective study	402 COVID-19 survivors (baseline) and 226 at 3-month follow-up (mean age 58.5 ± 12.8)	At hospital admission (baseline), 1 and 3 months after discharge	Mental health status: unstructured interview (DSM-5), IES-R, PCL-5, ZSDS, BDI-13, STAI-Y, WHIMS, OCI	Mental health status: 8.8% of patients were diagnosed with major depressive disorder or anxiety disorders (DSM-5), while 28% had symptoms of depression on the ZSDS index (≥ 50). Those with a previous psychiatric diagnosis were at higher risk of a major psychiatric disorder after COVID-19 infection (χ² =20.12; p < 0.001). Depressive symptomatology persisted between the 1-month and 3-month follow-ups, while PTSD symptoms, anxiety, and insomnia reduced significantly. Predictors of depression at the 3-month follow-up were female sex (Wilks’λ = 0.92, F = 5.76, p = 0.003), psychiatric history (Wilks’λ = 0.93, F = 5.29, p = 0.006), and psychopathology at 1-month follow-up (Wilks’ λ = 0.82; F = 15.16; p < 0.001).
					Cognitive functions: BACS	Cognitive functions: the severity of depressive symptoms (ZSDS) had a negative effect on neurocognitive functioning at the 1 and 3-month follow-up. Specifically, depression predicted reduced information processing performance (1 month: Wald = 7.05, p = 0.007; 3 months: Wald = 8.37, p = 0.003).
					Inflammatory parameters: CRP, NLR, MLR, SII*	Inflammatory parameters: depression at 3-months follow-up was predicted by systemic inflammation (SII level) at baseline: (ZSDS: χ² = 42.417, p < 0.0001; BDI-13: χ² = 56.536, p < 0.0001) and changes of SII (ZSDS: Wald W² = 6.881, p = 0.0087; BDI-13: Wald W² = 14.304, p = 0.0002).
Yang et al.^23^	Tongji Medical College, Huazhong University of Science and Technology, Wuhan, □Hubei□, □China	Single-center prospective study	28 recovered COVID-19 patients (mean age 40 ± 7.9) and 27 healthy controls (37.7 ± 9.0)	Three months after hospital discharge	Mental health status: PHQ-9, GAD-7, PTSD-SS, PTSD PCL-C, HAMA, HAMD	Mental health status: recovered COVID-19 patients showed significantly higher indices on the GAD-7, HAMA, HAMD, PTSD-SS, and PCL-C.
					Cerebral white matter imaging: magnetic resonance imaging (Siemens 3 T scanner, Magnetom Skyra, Siemens, Erlangen, Germany)	Cerebral white matter imaging: recovered COVID-19 patients showed decreased fractional anisotropy, increased mean diffusivity, and radial diffusivity values in multiple white matter tracts.
					Plasma untargeted metabolomic: LC-MS analysis (ultra-high-performance liquid). chromatography (Ultimate 3000), system coupled to a Q Exactive HRM system (Thermo Scientific, USA)	Plasma untargeted metabolomic: 22 metabolites (including xanthosine, hypoxanthine, and arachidonic acid) were significantly elevated and 30 metabolites (including taurine, serotonin, inosine, and adenosine) were reduced in recovered COVID-19 patients compared to healthy controls.
The PHOSP-COVID Collaborative Group, 2022^24^	83 hospitals across UK	Multicentric prospective study	2,320 patients discharged from hospital (mean age 58.0 ± 12.6), out of which 807 assessed at 5- and 1-year follow-up (mean age 58.7 ± 12.5)	Three months and 1 year after hospital discharge	Mental health status: GAD-7, PHQ-9, PCL-5	Mental health status: cognitive impairments were associated with trait anxiety and depression
					Cognitive functions: MoCA	Cognitive functions: after 1 year, 8.8% had significant cognitive impairment. However, it significantly improved at 1 year in the moderate with cognitive impairment cluster and was unchanged in the other clusters.
					Inflammatory parameters: the Olink Explore 384 Inflammation panel	Inflammatory parameters: The following proteins were significantly increased in participants in the very severe recovery cluster compared with those in the mild cluster: trefoil factor 2, transforming growth factor a, lysosomal associated membrane protein 3, CD83 molecule, galectin-9, urokinase plasminogen activator surface receptor, IL-6, erythropoietin, FMS-related receptor tyrosine kinase 3 ligand, agrin, secretoglobin family 3A member 2, follistatin, and C-type lectin domain family 4 member D. IL-6 and CD70 molecule were increased in the moderate with cognitive impairment cluster compared with the mild cluster.

BDI-13 = 13-Item Beck Depression Inventory; COVID 19 = coronavirus disease 2019; DSM-5 = Diagnostic and Statistical Manual of Mental Disorders, 5th edition; GAD-7 = Generalized Anxiety Disorder 7-Item Scale; HAMA = Hamilton Anxiety Rating Scale; HAMD = Hamilton Depression Rating Scale; HRM = high-resolution mass spectrometry; IES-R = Impact of Event Scale-Revised; IL = interleukin; LC-MS = liquid chromatography-mass spectrometry; MoCA = Montreal Cognitive Assessment; OCI = Obsessive-Compulsive Inventory; PCL-5 = Post-Traumatic Stress Disorder Scale (PTSD) Checklist for DSM-5; PCL-C = PTSD Checklist-Civilian Version; PHOSP-COVID = Post-Hospitalization COVID-19 Collaborative Group; PHQ-9 = Patient Health Questionnaire-9; PTSD-SS = PTSD Self-Rating Scale; STAI-Y = State-Trait Anxiety Inventory Form Y; WHIMS = Women's Health Initiative Insomnia Rating Scale; ZSDS = Zung Self-Rating Depression Scale.

* Platelets × neutrophils/lymphocytes.

### Neuroendocrine/metabolic dysfunction

Yang et al.^23^ studied the relationship between structural changes in cerebral white matter, mental health status, and metabolism in recovered COVID-19 patients. Twenty-eight (n = 28) recovered COVID-19 patients, 3 months after hospital discharge with no previous underlying diseases, were compared with 27 healthy controls. A mental health evaluation found significantly higher scores in recovered COVID-19 patients on the Post-Traumatic Stress Disorder Self-Rating Scale^25^ (PTSD-SS) (mean 29.5; standard deviation [SD] 26.5-38 vs. 24; SD 24-25, p < 0.01), the PTSD Checklist-Civilian Version^26^ (PCL-C) (23; SD 19-27.5 vs. 18; SD 17-18.5, p < 0.01), the Generalized Anxiety Disorder Screener^27^ (GAD-7) (1.5; SD 0-4 vs. 0; SD 0-1.5, p < 0.05), the Hamilton Anxiety Rating Scale^28^ (HAMA) (2.5; SD 1-5.25 vs. 1; SD 0-3, p < 0.05), and the 17-item Hamilton Depression Rating Scale^29^ (HAMD) (5; SD 2-9.25 vs. 2; SD 1-4.5, p < 0.01). Moreover, several metabolites, including xanthosine, hypoxanthine, and arachidonic acid, were significantly elevated, while others, including taurine, serotonin, inosine, and adenosine, were significantly reduced in the peripheral blood of recovered COVID-19 patients. These metabolic alterations were primarily related to purine pathways, amino acids, lipids, and amine metabolism. Magnetic resonance imaging revealed significant correlations between some of these metabolic alterations and changes in the white matter of specific brain regions. This included a positive association between arachidonic acid and left cingulum radial diffusivity (R = 0.415, p = 0.002), and serotonin with left anterior corona radiata (R = 0.308, p = 0.022). Amino acid alterations were significantly correlated with changes in the left cingulum, the left anterior corona radiata, and the left internal capsule.^23^

### Inflammation

A prospective cohort study was conducted in Italy with 402 COVID-19 survivors, who were followed up 3 months after hospital discharge.^11^ Unstructured interviews were conducted using Diagnostic and Statistical Manual of Mental Disorders, 5th edition (DSM-5) criteria to identify current mental disorders. Psychopathology was also assessed after 1 and 3 months using the following validated self-reported questionnaires: the Impact of Event Scale-Revised (IES-R),^30^ the PTSD Checklist for DSM-5 (PCL-5),^31^ the Zung Self-Rating Depression Scale (ZSDS),^32^ the 13-item Beck Depression Inventory (BDI-13),^33^ the State-Trait Anxiety Inventory Form Y (STAI- Y),^34^ the Women's Health Initiative Insomnia Rating Scale (WHIIRS),^35^ and the Obsessive-Compulsive Inventory (OCI).^36^

Of the 402 patients assessed 1 month after hospital discharge, 226 were assessed again at a 3-month follow-up (age 58.5 ± 12.8; range 26-87 years). Of these, 35% (n = 81) scored in the clinical symptomatology range on at least one of the four psychopathological dimensions (depression, anxiety, PTSD, and obsessive-compulsive disorder). Higher scores were observed in women, those with a psychiatric history, and those who had shown psychopathology at the 1-month follow-up. In the unstructured clinical interview, 24.3% met the diagnostic criteria for at least one mental disorder: major depressive disorder (n = 20), anxiety disorders (n = 20), insomnia (n = 7), and other diagnoses (n = 8). A comparison of the assessments at the 1- and 3-month follow-ups found reductions in anxiety symptoms (STAI-state: F =11.28, p = 0.001), PTSD (IES-R: F = 21.29, p = 0.001; PCL-5: F = 9.07, p = 0.003), and insomnia (WHIIRS: F = 9.36, p = 0.003). However, there was no change in depression scores (ZSDS and BDI), regardless of gender or psychiatric history.^11^

At hospital admission (baseline), inflammatory markers were measured. These comprised C-reactive protein (CRP), the neutrophil/lymphocyte ratio (NLR), the monocyte/lymphocyte ratio (MLR), and the systemic immune-inflammation index (SII) (SII = platelets × neutrophils/lymphocytes). In a subgroup of 45 patients, these markers were also assessed at the 3-month evaluation. Interestingly, the SII predicted the severity of depressive symptomatology at the 3-month follow-up, and inpatients – who had higher SII levels than outpatients – scored higher on the depression scales, after correcting for age and sex. Reductions in inflammatory markers 3 months after discharge were correlated with lower depression scores on both the BDI-13 (Wald W² =14.304, p = 0.0002) and the ZSDS (Wald W² = 6.881, p = 0.0087), controlling for age, sex, and hospitalization. Reduced expression of inflammatory biomarkers was correlated with reductions in depressive symptoms, while those with only slight changes in SII levels had persistent or worsening depressive symptoms.^11^

Cognitive functions were assessed using the Brief Assessment of Cognition in Schizophrenia (BACS)^37^ in a subsample of 130 patients under 70 years old at the 1- and 3-month follow-ups. In cognitive assessments, 81% of patients (n = 105) had scores below the normal range in at least one domain, and this had significant effects on psychopathology at one (Wilks’ λ = 0.81; F = 3.39; p = 0.004) and 3 months (Wilks’ λ = 0.82; F = 2.32; p = 0.042) after discharge. At 1 month, average performance was worse in three domains: verbal fluency (ß = 0.349, p = 0.002), information processing (ß = 0.348, p = 0.002), and executive functions (ß = 0.353, p = 0.001). Only information processing was associated with psychopathology at the 3 months assessment (β = 0.355, p = 0.008). Information processing performance was predicted by the severity of depressive symptoms at both the 1 (Wald = 7.05, p = 0.007) and 3-month follow-ups (Wald = 8.37, p = 0.003). Moreover, SII at hospital admission predicted neurocognitive performance. Specially, a significant negative main effect of age on all measures of neurocognitive performance was found, as well as a significant interaction between SII and age on measures of verbal memory (χ² = 4.908, p = 0.0267), verbal fluency (χ² = 4.273, p = 0.0387), speed of information processing (χ² = 5.544, p = 0.0185), and psychomotor coordination (χ² = 6.680, p = 0.0097).^11^

The Post-Hospitalization COVID-19 (PHOSP-COVID) Collaborative Group conducted a multicentric cohort in 83 hospitals across the UK and sought to understand the participants’ inflammatory profiles and other factors associated with health status and patients-perceived recovery 1 year after discharge from hospital by COVID-19.^24^ Recovery was stratified by both patient-perceived recovery and clusters of recovery (very severe, severe, moderate with cognitive impairment, and mild), defined 5 months after hospital admission based upon severity of current physical status, mental health, and cognitive impairment.

Overall, after 1 year of discharge, only 28.9% of 804 patients self-perceived fully recovered and 392 (48.8%) were not recovered. The distribution of the four clusters was as follows: very severe physical and mental health impairment (n = 319 [19.5%]), severe physical and mental health impairment (n = 493 [30.1%]), moderate physical health impairment with cognitive impairment (n = 179 [10.9%]), and mild mental and physical health impairment (n = 645 [39.4%].^24^

Three factors were associated with a lower probability of recovery: 1) female sex (odds ratio [OR] = 0.68; 95%CI 0.46-0.99); 2) body mass index (BMI) ≥ 30 kg/m² (0.50; 95%CI 0.34-0.74); and 3) receiving invasive mechanical ventilation (0.42; 0.23-0.76). Interestingly, proportions of women and participants with obesity were higher in the very severe cluster than the mild cluster.^24^

Among several outcome measures, mental health was assessed by the GAD-7,^27^ the Patient Health Questionnaire-9 (PHQ-9),^38^ and the Post Traumatic Stress Disorder Checklist (PCL-5).^36^ After 1 year, 147 (21.5%) patients had clinically relevant symptoms of anxiety, 169 (24.9%) had clinically relevant symptoms of depression, and 68 (10%) had PTSD. In the very severe cluster, symptoms of depression and anxiety significantly improved between 5 months and 1 year.^24^

Additionally, cognitive function was evaluated by the Montreal Cognitive Assessment (MoCA).^39^ At the 1 year-follow up, 55 (8.8%) had significant cognitive impairment. Cognitive impairment significantly improved at 1 year in the moderate with cognitive impairment cluster and remained unchanged in the other clusters.^24^

The inflammatory profile was evaluated through biochemical tests. Overall, after adjustments for confounders, several proteins – including interleukin (IL)-6 – were significantly increased in participants in the very severe recovery cluster compared with those in the mild cluster. Additionally, IL-6 and the CD70 molecule were significantly increased in the moderate with cognitive impairment cluster compared with the mild cluster. Moreover, after 1 year of discharge, the very severe cluster was associated with greater rate of participants with increased CRP concentration > 5 mg/L compared with the mild cluster (38.4% vs. 14.5%).^24^

## Discussion

The present review synthesizes the findings of three prospective longitudinal studies on the long-term mental health and cognitive sequelae of COVID-19 infection and their association with inflammation and metabolic markers. Following 3 months of COVID-19 infection, individuals who were female, had a history of psychiatric illness, and exhibited psychopathology after 1 month had higher scores in psychopathological dimensions, with depression persisting as a prominent symptom. Additionally, SII at the onset of the infection predicted the severity of depressive symptomatology and neurocognitive performance. While reductions in inflammatory markers were correlated with lower depression scores, cognitive impairment was independent of disease severity and affected 81% of patients, mainly in executive functions.

Moreover, patients’ self-perception of recovery after 1 year of hospital discharge remained poor, with less than a third of individuals showing full recovery and almost half reporting no recovery. Significant cognitive impairment persisted in 8.8% of patients, with the presence of inflammatory markers, female sex, and obesity associated with more severe clusters in self-perceived mental health, cognition, and physical health.

Finally, the metabolic profiling of plasma in recovered COVID-19 patients continued to differ from that of healthy controls even 3 months after hospital discharge. These changes, associated with compromised white matter integrity, were correlated with mental health scores, indicating the potential involvement of metabolic dysregulation in the persistent psychiatric symptoms following COVID-19 infection.

It is well known that COVID-19 infection increases the risk of mental disorders’ incidence. A large 1-year cohort study conducted in the United States assessed the risk of COVID-19 effects on mental health.^40^ The authors compared patients (n = 153848) after 30 days of COVID-19 infection with three different groups. The COVID-19 group was found to have a significantly higher incidence of anxiety disorders than a healthy, contemporary control group (n = 5807309; hazard ratio 1.35, 95%CI 1.30-1.39), depressive disorders (1.39 (1.34-1.43)), and stress and adjustment disorders (1.38 (1.34-1.43)). When compared with a historical group from the pre-pandemic period and a control group of influenza patients after 30 days of infection, the COVID-19 group had an increased incidence in all of the pre-specified outcomes analyzed.^40^

**Figure 1:**
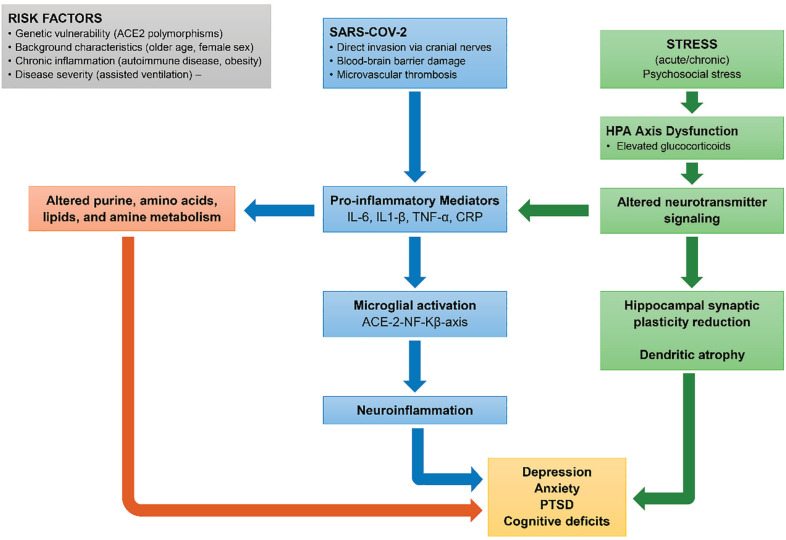
Pathophysiological mechanisms of depression, anxiety, and cognitive deficits in COVID-19. ACE2 = angiotensin-converting enzyme 2; CRP = C-reactive protein; HPA = hypothalamic-pituitary-adrenal; IL = interleukin; NF = nuclear fator; PTSD = post-traumatic stress disorder; SARS-CoV-2 = severe acute respiratory syndrome coronavirus 2; TNF = tumor necrosis factor.

Building upon the primary findings of this review, we will explore the pathophysiological changes associated with COVID-19, including inflammation, metabolomics, cognition, and stress. Figure 1 displays the pathophysiology of depression, anxiety, and cognitive deficits in COVID-19.

### The pathophysiology of inflammation in COVID-19 and its relation to psychiatric symptoms

COVID-19 infection begins when the virus enters the body through the nasal cavity and pulmonary alveoli and infects cells through the angiotensin-converting enzyme-2 (ACE-2) receptor. It then stays in the cellular cytoplasm and replicates. Innate immune activation results in the secretion of pro-inflammatory cytokines such as tumor necrosis factor (TNF)-α, IL-1beta, IL-6, and IL-18. The immune response of the host activates CD4+ and CD8+ T cells to control the infection.^41^

Interestingly, the severity of the infection seems to depend on both direct viral toxicity and how quickly the host produces specific viral antibodies and activates T cells. When there is a rapid and effective T cell response, COVID-19 infection is controlled, resulting in mild or asymptomatic disease. However, a delay in the immune response (i.e., a delay in type I and III IFN responses) can result in excessive viral replication and its spread in the target organs (i.e., the lungs). This leads to excessive and uncontrolled release of pro-inflammatory cytokines, a process known as a cytokine storm.^41^ This seems to be the main cause of mortality from the disease, as it can lead to respiratory failure and multiorgan dysfunction.^42^ A retrospective study of 452 COVID-19 patients in Wuhan, China, compared severe and non-severe cases. Severe cases showed more infectious markers (i.e., procalcitonin, erythrocyte sedimentation rate, serum ferritin, and CRP) and pro-inflammatory cytokines (i.e., TNF-α, IL-2R, and IL-6).^43^ Another study of 1484 patients in New York, USA found that elevated levels of IL-6 and TNF-α were strong and independent predictors of disease severity and mortality, even after adjusting for disease severity, inflammatory markers, hypoxia, and comorbidities.^44^ The results of the cohorts included in this review shed light on this subject, by demonstrating associations between inflammatory markers and mental health disorders.

It is of note that the host immune response to COVID-19 infection has systemic repercussions, which may result in changes in the CNS (i.e., blood-brain barrier disruption, neurotransmission alterations, microglial activation, and oxidative stress) associated with depression.^45^ These changes could be due to direct infection of the brain, autoantibody formation, or distal inflammatory responses. A recent study suggests that the SARS-CoV-2-spike glycoprotein can activate microglia through the mediation of the ACE-2-NF-kB axis.^46^ This mechanism may promote activation of the microglial NLRP3 inflammasome and lead to neuroinflammation and neurological manifestations, such as Parkinson's disease. Additionally, several studies have found that autoimmune diseases (i.e., rheumatoid arthritis, asthma, lupus) and pro-inflammatory conditions such as obesity increase the risk of depression. Furthermore, individuals with depression, generalized anxiety disorder, and PTSD have higher levels of pro-inflammatory markers (i.e., CRP, IL-1, IL-6, TNF-α) compared to healthy controls.^47^ IL-6, in particular, appears to play a central role in the relationship between depression and long-term COVID, producing dysregulation between TH17/Treg lymphocytes and sending signals to brain regions implicated in depression such as the anterior cingulate cortex.^48^

The PHOSP-COVID Collaborative Group study reveals that long-lasting systemic inflammation negatively impacts both the mental and physical health of people infected with COVID-19.^24^ Those patients belonged to the very severe cluster and tended to present more obesity, lung damage, and a range of elevated inflammatory markers than the mild cluster. IL-6 was particularly higher in both the very severe and the moderate with cognitive impairment groups, than the mild group. These results are reinforced by the study of Mazza et al.,^11^ which revealed that changes in inflammatory markers correlated with changes in depressive symptomatology. Taken together, both studies reinforce the idea of the interplay between the inflammatory responses induced by COVID-19 infection and mental disorders, which may reinforce one another, resulting in increased inflammation and worse the symptomatology. Moreover, they point to the possibility of using anti-inflammatory agents as pharmacological targets as well as multidisciplinary approaches that could ameliorate physical and mental health symptoms, including cognitive rehabilitation and the promotion of healthy lifestyle habits.

### Neuroendocrine/metabolic dysfunction in COVID-19

The COVID-19 infection induces systemic metabolic shifts in multiple molecular phenotypic states. These changes, known as "phenoconversion", have been studied through mass spectrometry and nuclear magnetic resonance spectroscopy to identify patterns of metabolites associated with COVID-19. This research provide insights into the underlying pathophysiological processes and their individual variations, as well as their relationships to disease severity and/or recovery.^2,49-52^ Protein and metabolite changes in the sera of COVID-19 patients have been demonstrated, which implicates dysregulation of macrophages, platelet degranulation, complement system pathways, and massive metabolic suppression.^49^ The serum metabolite profile of COVID-19 patients can take up to 10 months to return to normal levels, despite clinical indicators remaining within the normal range.^53-55^ Post-COVID-19 metabolic disorders have been observed in recovered moderate and critical patients^54^ and even subsequent to asymptomatic or mild COVID-19 cases.^55^

Serum metabolic profiling can detect biological states related to mental disorders and can identify potential blood-based biomarkers of psychiatric diseases.^56^ In the Yang et al.^23^ study, the alterations found in purine pathways in recovered COVID-19 patients could give a clue for possible pathophysiological mechanisms underlying the effects on mental health of COVID-19. Xanthine, one of the compounds of the purine pathway, acts on the amygdala, which plays a critical role in generating fear and anxiety. Arachidonic acid was significantly elevated in the same study, and this may provide insights into the role of lipid metabolism in the mental health effects of COVID-19. Fatty acids are necessary for the function of all cells, especially in the nervous and immune systems, and metabolites of the arachidonic acid pathway are known to affect the nervous system, contributing to symptoms of anxiety.^57,58^

Additionally, Yang et al.^23^ found reductions in serotonin and taurine in recovered COVID-19 patients. Serotonin dysregulation is implicated in multiple emotional disorders, including anxiety and depression.^59,60^ Taurine, in turn, is known to prevent oxidative stress and inflammation and hence acts as an endogenous neuroprotector.^61^

Moreover, lower serum tryptophan levels and activation of the kynurenine pathway have been documented in depression^62-64^ and a possible association between tryptophan-kynurenine disturbance and long COVID-19 has been postulated to explain the development of post-COVID depression.^65-67^ Tryptophan is an essential amino acid that is converted into either serotonin or kynurenine. The process that diverts tryptophan toward kynurenine involves indoleamine 2-3-dioxygenase (IDO) and tryptophan 2,3-dioxygenase^68,69^ and this process has the potential to promote depression.

Lionetto et al.^70^ compared tryptophan and kynurenine serum levels by high performance liquid chromatography—tandem mass spectrometry (HPLC/MS-MS) in COVID-19 patients to healthy controls. Those with COVID-19 showed increased kynurenine: tryptophan ratios and the level of increase was positively correlated with the severity of infection. Targeted and untargeted metabolomic analyses have also identified altered tryptophan metabolism in the kynurenine pathways of COVID-19 patients.^71,72^ These metabolic alterations are thought to result from cytokine storms that activate the enzyme IDO-1, increasing kynurenine metabolites. This, in turn, may lead to long-term cognitive impairment and mental illness, such as depression.^73,74^

Another proposed mechanism for the monoamine synthesis disturbances that may exacerbate or trigger mental health disorders is the disruption of angiotensin-converting enzyme 2 (ACE2) in the gastrointestinal tract. The gastrointestinal symptoms of COVID-19 and viral presence in stool suggest enteric invasion, and ACE-2 in the gastrointestinal tract can be affected. Enteric ACE-2 is responsible for regulating the uptake of amino acids, including tryptophan. Thus, impaired tryptophan uptake and the subsequent reduction in serotonin, dopamine, and melatonin may contribute to the impairment of mood, memory, and sleep associated with COVID-19.^75^

Finally, significant correlations were found between amino acid alterations and changes in the left cingulum, a bundle of axons that facilitates communication between various areas of the limbic system. This region is believed to play a crucial role in the regulation of emotions, and changes to it could potentially contribute to the development of anxiety and PTSD.^23^

### Cognitive impairment induced by COVID-19

It has been suggested that the occurrence of cognitive and mental health dysfunctions in recovered COVID-19 patients may be bidirectional in nature. A systematic review found that the most prevalent neuropsychiatric signs and symptoms reported in post-illness studies included sleep disorders (100%), recall of traumatic memories (30.4%), emotional lability (23.5%), impaired concentration or attention (19.9%), and impaired memory (18.9%).^12^ In the acute phase, attentional (38.2%) and memory (34.1%) impairments, insomnia (41.9%), anxiety (35.7%), and depressed mood (32.6%) prevail. The severity of depressive symptoms seems to predict the extent of cognitive deficits, especially the information processing. The degree to which psychopathology, cognitive impairments, and disease severity are associated with the development of long COVID-19 has not yet been fully established.

Two studies included in this review indicate that the SARS-CoV-2 infection seems to affect goal-directed cognitive skills that rely mainly on pre-frontal functioning.^11,24^ The PHOSP-COVID study^24^ found that, among the 55 (8.8%) participants who had significant cognitive impairment at 1 year after discharge, slowing down in thinking and short-term memory loss were the most reported. The cognitive deficits reported by Mazza et al.^11^ affected mainly verbal fluency, information processing and executive functions and were predicted by inflammatory markers at the beginning of the COVID-19 infection.

The persistent cognitive impairments after SARS-CoV-2 infection seem to be related to systemic inflammation, as it happens to depression. The underlying mechanism may be a cytokine imbalance.^76^ The increase of IL-6, TNFα, and IL-1β cytokines, which is related to working memory and attention impairments, are also common in cognitive deficits observed in delirium states. Moreover, a recent study demonstrated that the chemokine CCL11, associated with impairments in cognitive function and neurogenesis, remains elevated in long COVID-19 patients experiencing cognitive symptoms compared to those without cognitive symptoms, suggesting an inflammatory response contributing to cognitive deficits.^9^ Thus, cytokines may become useful as inflammatory markers for cognitive impairments after SARS-CoV-2 infection.

### Pathophysiology of stress induced by COVID-19

The COVID-19 pandemic can have consequences on mental health and cognition due to continuous exposure to stress, fear of the unknown, prolonged social distancing, and economic problems.^77^ A study evaluated neuropsychiatric and functional changes in 58 outpatients with dementia through the perceptions of their caregivers during the pandemic.^78^ The study found that over half of the patients experienced cognitive decline, almost half experienced behavioral symptoms including apathy/depression, and over a third experienced functional decline. These findings suggest that the pandemic and social isolation measures have had a significant impact on the neuropsychiatric symptoms of dementia patients and their caregivers’ burden.^78^

The acute stress in response to the aversive COVID-19 pandemic environment initially activates the hypothalamic-pituitary-adrenal axis, increasing the release of cortisol by the adrenal cortex. When the cortisol interacts with glucocorticoid receptors in the frontal cortex and the hippocampus, these receptors operate as transcription factors, modeling the neural circuitry that regulates cognition and emotional response to stress.^79^ In chronic stress, excess of glucocorticoids results in shrinking of dendritic processes and impairment of hippocampal synaptic plasticity, which can impair cognitive functions and emotions, increasing the risk of depression.^79^

Moreover, exposure to traumatic situations leads to changes in inflammatory markers associated with PTSD, including elevations in the CRP and the chemokines CCL13, CCL20, and CXCL6, and lowered levels of TNF-α and interferon-gamma.^32^ In this review, the PHOSP-COVID Collaborative Group found that 10% of the individuals discharged from the hospital due to COVID-19 presented PTSD diagnoses after 1 year.^24^

This review has several limitations. Firstly, the limited number of studies included may not provide a comprehensive overview of the research in this area. Secondly, we may not be able to distinguish between high-quality and low-quality evidence, which could have introduced selection bias. Thirdly, we may have been subject to publication bias by including only studies that reported statistically significant results. Finally, the included studies have their own limitations that should be considered before reaching any conclusions.

## Conclusion

This review presents evidence of the persistent psychiatric and cognitive symptoms following COVID-19 infection. Together, these prospective longitudinal studies suggest the complex and long-term impacts of COVID-19 on mental health and cognition, and the potential involvement of inflammation and metabolic dysregulation in these persistent symptoms.

Long COVID-19 has become a global public health issue. Therefore, longitudinal studies with large samples are critical if we want to disentangle the roles of the metabolic and inflammatory alterations involved in psychiatric symptoms and cognitive impairments caused by COVID-19. Additionally, metabolomics can make vital contributions to predicting outcomes for COVID-19 patients with a greater degree of certainty.
